# Giant Ovarian Cyst Masquerading as Massive Ascites in an 11-Year-Old

**DOI:** 10.1155/2015/878716

**Published:** 2015-07-05

**Authors:** Shaza Ali Mohammed Elhassan, Shabina Khan, Ahmed El-Makki

**Affiliations:** Hamad Medical Corporation, P.O. BOX 3050, Doha, Qatar

## Abstract

We are presenting a unique case of an 11-year-old girl admitted for investigation of progressive abdominal distention of more than one-year duration. Due to the complete cystic nature of the mass and its enormous size, it was not visualized by the ultrasound and was reported as massive ascites. MRI and postoperative histopathology confirmed a diagnosis of giant serous cystadenoma of the right ovary. She underwent a right ovarian cystectomy with complete preservation of both ovaries and fallopian tubes and is doing well on outpatient follow-up.

## 1. Introduction

Although an ovarian cyst detected in an adolescent girl is most likely a physiological or functional cyst [1], it still deserves a thorough evaluation as up to 15% of all cystic ovarian lesions prove to be neoplastic [2, 3]. Neoplastic lesions of the ovary are classified based on the anatomic tissue from which they originate—germ cell tumors, epithelial cell tumors, and stromal cell tumors [4]. The epithelial cell tumors (serous or mucinous cystadenomas) are most commonly encountered in the fourth or fifth decade of life, but they should also be considered to be differential diagnoses in pediatric patients given that they are the second most common benign ovarian tumor in adolescents [4]. These tumors can reach giant proportions and very few cases of giant serous cystadenomas in adolescents have been reported. We present one such case of an 11-year-old girl who was admitted to our hospital for investigation of progressive abdominal distention of one-year duration. Due to the complete cystic nature of the mass and its enormous size, it was not visualized by the ultrasound and was reported as massive ascites. MRI and postoperative histopathology confirmed a diagnosis of serous cystadenoma of the right ovary.

## 2. Case Report

Our case is an 11-year-old Sudanese girl, residing in Qatar, who presented to the pediatric outpatient department with an 18-month history of progressive abdominal distention. The parents had been attributing the abdominal distention to weight gain, especially as the child remained otherwise asymptomatic. They had in fact been encouraging the girl to lose weight. The parents sought medical advice at a local health center, due to flu-like symptoms. The examination at the health center was impressive for signs of massive abdominal distention and she was admitted to our tertiary care hospital promptly for further investigations with a preliminary diagnosis of ascites. Apart from the progressive abdominal distention, there was no other contributory history suggestive of any underlying malignancy, liver disease, heart failure, or undiagnosed renal problems. The girl did not complain of any abdominal pain, constipation, urinary retention, or respiratory distress secondary to her abdominal distention. Our patient had achieved menarche 1 year ago with infrequent menstrual cycles; her first day of the last menstrual period was two weeks prior to her admission. There was no history of menorrhagia or dysmenorrhea.

Upon examination, the child was noted to be in good general condition with normal vital signs for age and in no apparent pain or distress. Her weight was 64.5 kg, which was above the 95th centile for her age. Apart from the massive abdominal distention, she was thin built. Her general examination did not reveal any clubbing, pallor, icterus, peripheral edema, or lymphadenopathy. There were no stigmata of chronic liver disease. Abdominal examination revealed a huge uniformly distended abdomen (maximum diameter was 105 cm), extending from the pelvis to the xiphisternum with full flanks. There were no visible dilated veins on the abdomen. Palpation did not reveal any tenderness or masses; fluid thrill was positive. She had normal female genitalia. Her respiratory, cardiovascular, and nervous system examinations were unremarkable. A bedside urine dipstick did not reveal any proteinuria.

As the physical findings detected a fluid thrill, her preliminary investigations were directed towards finding a likely explanation for what seemed like a massive ascites. Her preliminary laboratory work-up which included a complete blood count, peripheral smear, serum electrolytes, renal and liver functions tests were within normal. As our patient did not show any signs of chronic liver or renal disease, there was a strong concern among the treating physicians that the presumed ascites could be secondary to an underlying abdominal malignancy. At this stage, the pediatric oncology team was consulted and tumor markers which included Ca125 (6 U/mL), CEA 0.7 microgram/L, alpha-fetoprotein (<1.7 IU/mL) and beta-hCG (<5 IU/L) along with Uric acid 297 micromol/L, and LDH (174 U/L) were ordered, all of which were within normal limits.

**Figure 1:**
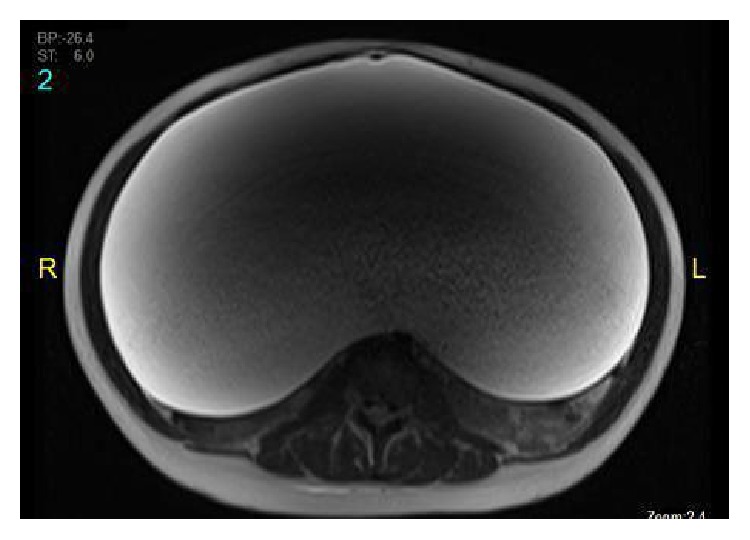
Abdominal ultrasound.

An urgent transabdominal ultrasonogram of the abdomen confirmed the suspicion of massive ascites (Figure 1). The possibility of requiring a diagnostic paracentesis was discussed with the family once the MRI of the abdomen and pelvis reasonably ruled out any underlying malignancy.

**Figure 2:**
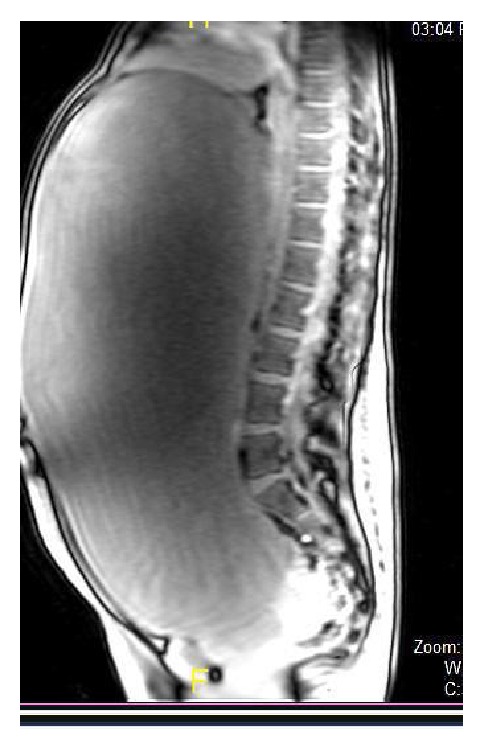
MRI abdomen.

The MRI of the abdomen and the pelvis (Figure 2) revealed that what was visualized as massive ascites by the sonographer was in fact a large homogenous well defined unilocular huge cystic abdominopelvic mass which measured 39 × 29 × 18 cm in dimension, occupying the entire abdomen and pelvis and bulging into the anterior abdominal wall. No solid component could be noted within the mass lesion. No loculation or septation was seen given the likelihood of serous cyst adenoma of the right ovary.

A lower abdominal midline incision was made revealing the peritoneum. An elliptical incision is carefully made through the ovarian cortex to the cyst wall.

**Figure 3:**
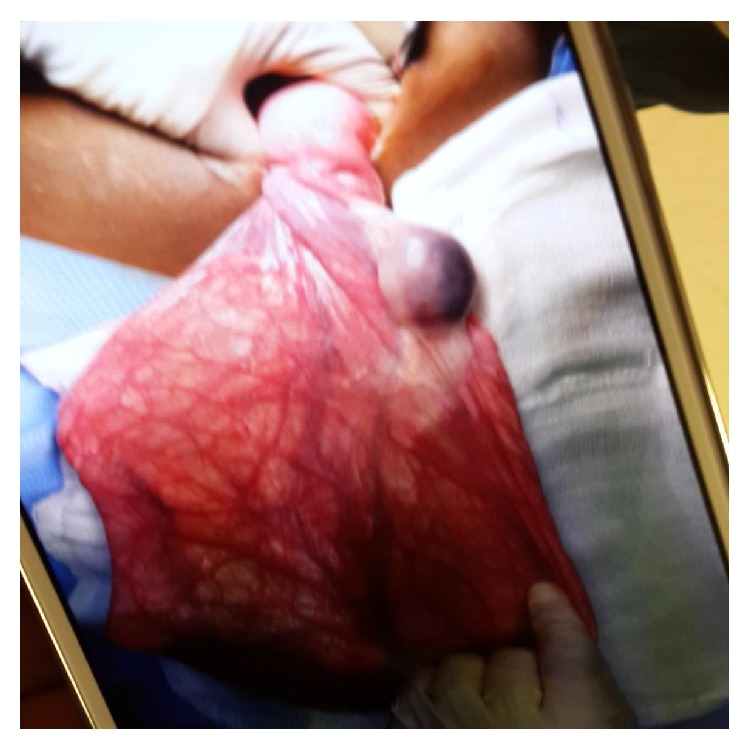
Intraoperative cystectomy.

When the cyst wall was reached, blunt and sharp dissection using surgical scissors was used to separate the cyst wall from the surface of the ovary. Intraoperative visualization did not reveal any abnormality of the left adnexal structures. The cyst was aspirated prior to its delivery and gave 13000 milliliters of fluid. The patient underwent right ovarian cystectomy with complete preservation of both ovaries and fallopian tubes. It weighed 13 kg and contained 13 liters of fluid. (Figure 3) Histopathological examination of the cyst revealed simple tubal-type epithelium confirming the diagnosis of a serous cystadenoma of the right ovary, consistent with the preoperative MRI diagnosis.

Our patient did well after surgery and was discharged on the fourth postoperative day. Her discharge weight was 48 kg. Upon follow-up a week after her surgery she showed an excellent recovery and will continue to have regular follow-up in our outpatient clinic with an ultrasound examination every three months for early detection of any recurrence.

## 3. Discussion

An ovarian mass in pediatrics may represent the commoner physiological functional ovarian cyst or be a benign or rarely malignant tumor [1]. The most common ovarian tumors encountered in pediatric practice are germ cell tumors, which account for about two-thirds of ovarian tumors in this age group [5]. Surface epithelial tumors including serous cyst adenomas are rare in pediatrics [4] with a reported international incidence of around 15–20% of all pediatric ovarian masses [6]. Compared to their adult counterparts, fortunately, 90% of ovarian masses seen in the pediatric and adolescent population are benign [7]. Differential diagnoses of ovarian masses in adolescence include cyst formation, ovarian torsion, benign or malignant ovarian neoplasm, and involvement of the ovary in lymphoma, leukemia, or metastatic disease [8].

As general pediatricians, we need to acknowledge that ovarian masses in general in our patient population are by no means uncommon [9] and can have a varied presentation. They may present as vague abdominal pain, acute abdomen, and an asymptomatic pelviabdominal mass, with features of hormonal derangement, or be discovered incidentally on a routine imaging [2]. On the other hand, ovarian tumors that reach giant proportions of greater than 15 cm are quite rare in this population [2]. Our patient was diagnosed with a giant serous cyst adenoma, and to date very few such cases have been reported in literature in adolescents ranging from the age of 13 to 19 years [10–16]. Our patient, who achieved menarche at age 10, was 11 years at the time of diagnosis and perhaps represents one of the youngest cases of giant serous cyst adenoma reported. Postpubertal estrogen and progesterone hormone levels are postulated to play a role in the pathogenesis of surface epithelial tumors, which might explain why the reported cases are mostly 13 years or older [5].

Giant ovarian tumors can be present as asymptomatically increasing abdominal girth [2] or be accompanied by symptoms of nausea, vomiting, weight loss or increased urinary frequency, urinary retention, constipation, and dyspnea due to pressure effects [17]. Despite being asymptomatic, giant ovarian tumors have potential for serious complications such as torsion, suppuration, obstruction, and perforation necessitating urgent admission [18]. Ultrasonography is considered the initial imaging modality for ovarian masses [4].

Our patient is unique not only in terms of her age, but also as she posed a diagnostic challenge in many aspects. Firstly, she presented with a huge asymptomatic abdominal distention, which upon initial clinical assessment was presumed to be massive ascites. Moreover, ultrasound of the pelvis and abdomen, in our case, confirmed this clinical diagnosis of massive ascites without delineating a possible cause, necessitating an urgent MRI, which led to the final diagnosis of a giant ovarian mass. If management was undertaken in our patient on the basis of ultrasound diagnosis alone (namely, paracentesis for the presumed ascites), it may have led to erroneous transabdominal aspiration of the undiagnosed ovarian cyst. If paracentesis was undertaken in this patient it could lead to infection, bleeding, and increased peritoneal adhesion, thus making surgical cystectomy more challenging [19]. Ultrasound alone, being operator dependent, should perhaps be interpreted in caution in such patients in whom there is no clear diagnosis based on history and examination. Moreover, our case highlights the fact the pediatrician needs to entertain the diagnosis of a giant ovarian tumor early on in pediatric patients who present with a huge abdominal mass with a noncontributory history or exam, regardless of the rarity of the condition.

Giant ovarian tumors have become rare in current medical practice, as most cases are discovered early during routine check-ups. In our case, the patient's family did not seek medical advice for 1.5 years, as they assumed that the increased abdominal girth was due to weight gain and put her on an intense diet and exercise regime. The patient's weight at admission was 67 Kg and at one week postsurgery was 55 Kg. Teaching families and raising awareness are extremely important aspects in such cases.

As pediatricians are often the first physicians who encounter such cases, they should be aware of asymptomatic ovarian tumors as a differential diagnosis for massive abdominal distension, given that they are the largest tumors found in the human body [20].

If the pure cystic mass reaches an enormous size and the tumor markers are within normal limits, as in our patient, serous or mucinous cyst adenomas should be considered in the differential diagnosis in adolescent patient [2]. Management of these cases is usually by conservative surgery including cystectomy or unilateral salpingooophorectomy, which are adequate for benign lesions. After conservative surgery, the patients must be followed up carefully because some tumors recur, especially if not completely removed during surgery.

According to literature review by Patel et al. [2], 7 cases of giant cell serous cyst adenoma from case reports in adolescent ranging from 13 to 19 years, the maximum weight of 29 kg, most presented with increased abdominal girth but otherwise were asymptomatic between 6 months and 2 years. One case presented as acute abdomen and another with palpable abdominal mass with pressure symptoms, that is, nausea, vomiting, constipation, dyspnea, and abdominal distention. The mode of the treatment for these patients varied, three of them underwent cystectomy with complete preservation of the affected ovary and adnexal structures, the other three underwent cystectomy with removal of ovary, and the remaining one patient underwent open cystectomy with salpingectomy [10–16]. Our patient had fertility preserving surgery, that is, cystectomy with complete preservation of the ovaries, as it is the preferred modality of treatment in this age group. Management of this pathology and fertility-conserving treatment need careful follow-up because of the possibility of recurrence and malignant transformation.

## 4. Conclusion

As rare as giant ovarian tumors are, pediatricians should be aware of its presentation and should include ovarian masses in their differential diagnosis of abdominal distention. Pediatricians should raise families' awareness for seeking medical advice early in case of persistent abdominal distension. Ultrasound should not be the only imaging modality especially in case of massive ovarian cysts as it may mimic ascites. This case report emphasizes the paramount importance of considering ovarian masses in the differential diagnosis of a patient who has abdominal distention without symptoms or signs of liver, renal, or cardiac diseases. It is also vital to raise awareness among the population to seek medical advice as early as possible to avoid complications such as ovarian torsion, rupture, and eventually infertility in such young age group. Fertility-conserving treatments, as in our patient, need careful follow-up because of the possibility of recurrence in the remaining ovary or malignancy transformation.
